# New insights into posttranslational modifications of Hippo pathway in carcinogenesis and therapeutics

**DOI:** 10.1186/s13008-016-0013-6

**Published:** 2016-03-31

**Authors:** Mingjing He, Zhuan Zhou, Anil A. Shah, Yang Hong, Qianming Chen, Yong Wan

**Affiliations:** Department of Cell Biology, University of Pittsburgh School of Medicine and University of Pittsburgh Cancer Institute, Hillman Cancer Center, 5117 Centre Avenue, HCC2.6c, Pittsburgh, PA 15213 USA; State Key Laboratory of Oral Diseases, West China Hospital of Stomatology, Sichuan University, Chengdu, 610041 Sichuan Peoples’ Republic of China

**Keywords:** Hippo pathway, Posttranslational modifications, Phosphorylation, Ubiquitylation, Sumoylation, Acetylation, Methylation, Cancer and anti-cancer treatment

## Abstract

PTMs (posttranslational modifications) such as ubiquitylation, sumoylation, acetylation and protein methylation are pivotal modifiers that determine the activation, deactivation or subcellular localization of signaling proteins, facilitating the initiation, amplification and transduction of signaling. Accumulating evidence suggest that several key signaling molecules in Hippo signaling pathway are tightly regulated by various types of PTMs. Malfunction of these critical signaling modules such as YAP/TAZ, MAT1/2 and LATS1/2 due to deregulated PTMs has been linked to a variety of human diseases such as cancer. In this review article, we summarized the current understanding of the impact of PTMs in regulating Hippo signaling pathway and further discussed the potential therapeutic intervention from the view of PTMs and Hippo pathway.

## Background

The impact of Hippo signaling pathway has been linked to a variety of critical biological processes and human diseases, including organ growth control, stem cell function, tissue regeneration and tumor suppression. Signaling modules in the Hippo pathway are tightly regulated in order to orchestrate the organ size and cellular proliferation during the development as well as homeostasis. While regulation of Hippo cascade can be achieved at various levels such as gene replication, transcription and protein translation, increasing emerging evidence has drawn our attention to posttranslational modifications (PTMs). PTMs converts the properties of functional protein through a series manners, including proteolytic cleavage, the addition of a modifying group to one or more amino acid, which result in alteration of protein activity, subcellular localization, turnover, and interactions with partner proteins. Malfunction of PTMs in yes-associated protein (YAP)/TAZ, a critical signaling node in Hippo pathway, could directly drive tumor initiation and invasion. This review aims to summarize the recently expanded understanding of PTMs in regulating Hippo signaling pathway. We will discuss how dysfunction of Hippo cascade due to the deregulated PTMs contributes to tumorigenesis and the current strategies of anti-cancer therapeutics through targeting the Hippo signaling pathway.

### Overview of the Hippo pathway

#### Kinases cascade of Hippo pathway in mammals

The classic Hippo pathway reveals the pivotal roles of PTMs in biological processes, especially phosphorylation and ubiquitination. The core of the Hippo pathway comprises a highly conserved signaling module that functions similarly in mammals and in *Drosophila melanogaster.* Since the discovery of the serine/threonine kinases Mammalian Ste20-like kinases (MST1/2) and Large tumor suppressor (LATS1/2) in humans (known as the Hpo and Warts kinases in fruit flies respectively), many additional components of the Hippo pathway have been identified, and a complex signaling cascade has emerged which integrates multiple upstream inputs from the plasma membrane into the nucleus, including the scaffolding protein Salvador homolog 1 (SAV1; Salvador in fruit flies, which interacts with MST1 and MST2) and the scaffolding proteins MOB kinase activator 1A (MOB1A) and MOB1B (Mats in fruit fly, which interact with LATS1 and LATS2, respectively) [1].

When the Hippo pathway is on, MST1/2 phosphorylates LATS1/2 through their SARAH coiled-coil domains. It also phosphorylates MOB1, which enhances MOB1’s ability to bind and activate LATS1/2. The LATS1/2 phosphorylation of YAP/TAZ (Yki in fruit flies) is a key event in the canonical Hippo pathway [2]. LATS1/2 phosphorylates YAP at S127 and TAZ at S89 to enhance cytoplasmic retention of YAP/TAZ by increasing the interaction between YAP/TAZ and 14-3-3 protein. When the Hippo pathway is off, the final effectors of Hippo pathway—transcriptional co-activators YAP and TAZ—are translocated into nucleus and bind to DNA via forming complexes with transcriptional enhancer factor TEFs (TEAD1-TEAD4; scalloped in fruit flies). They also bind to other transcription factors such as SMADs, T-box transcription factor 5 (TBX5), RUNT-related transcription factor 1 (RUNX1) and RUNX2 as well as Tumor protein p73 to regulate gene expression. As the major downstream effector of the tumor suppressing Hippo pathway, YAP/TAZ were at first recognized as oncogenes. Elevated YAP/TAZ expression and nuclear localization have been observed in multiple types of human cancers, including which located in breast, lung, liver, colon, cervix, ovary, and esophagus [3–5]. However, recent data suggest an interesting hypothesis that YAP also has tumor suppressor function in certain contexts [6, 7].

#### Upstream regulators of Hippo pathway

**Fig. 1:**
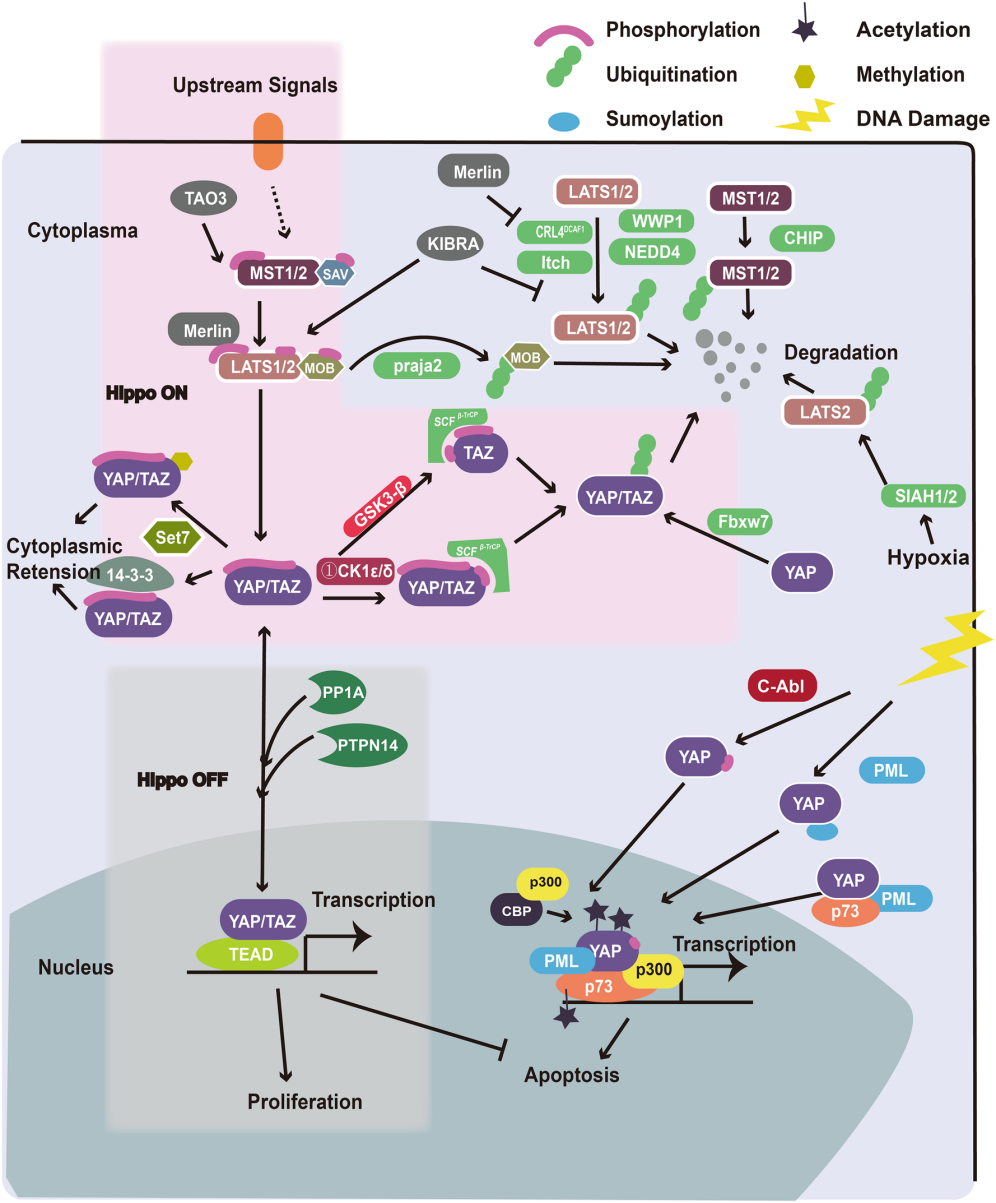
PTMs regulation of Hippo pathway. *Left top pink region* the canonical inhibitory kinase module of Hippo pathway. A kinase cascade comprising MST and LATS phosphorylates YAP and TAZ. The phosphorylated YAP and TAZ bind to 14-3-3 and are sequestered in the cytosol. Monomethylated YAP via Set7 is also critical for cytoplasmic retention. Merlin activates Hippo pathway via binding LATS and inhibiting CRL4DCAF1 E3 ubiquitin ligase. TAO3 activates MST by phosphorylation in the same sites as those autophosphorylation sites of MST. *Left bottom grey region* Proliferation is induced when Hippo pathway is off. YAP and TAZ are translocated into nucleus and bind to DNA via forming complexes with Transcriptional enhancer factor TEFs (TEAD1-TEAD4) and other transcription factors*. Left bottom region* YAP mainly mediates p73-dependent pro-apoptotic gene transcription in some situation such as DNA damage irritation. PML recruits YAP to the nuclear body and stabilizes p73. C-Abl promotes the association of YAP with p73, promoting apoptosis. ①NEK1/CK1ε/δ for TAZ

The Hippo pathway bears many upstream regulators that feed into the serine/threonine kinases MST1/2 and LATS1/2. PTMs regulation network is described in Fig. 1. Most well-known ones are Merlin, RAS association domain-containing family proteins (RASSFs) and protein kidney and brain expressed protein (KIBRA).

Merlin is a cytoskeletal protein and was identified as a tumor suppressor underlying Neurofibromatosis type II. Merlin contains a conserved FERM domain and was reported to be an upstream regulator of Hippo pathway [8]. Recently, Merlin was found to directly bind and recruit LATS to the plasma membrane and promote LATS phosphorylation via the MST-SAV kinase complex, suggesting an important role of Merlin in activating the Hippo-pathway [9].

In Drosophila, dRASSF competes with Sav for Hpo and recruits a PP2A complex (dSTRIPAK) to dephosphorylate and inactivate Hpo [10]. However, multiple RASSF isoforms in mammals showed different roles on the Hippo pathway, suggesting a divergent role through evolution. RASSF6 inhibited MST2 and antagonized Hippo signaling in a manner similar to that of dRASSF [11]. RASSF1 and RASSF5 stimulate Hippo signaling and play a distinct role from dRASSF. They bind the MST and counteract dephosphorylation, then activate NDR1, NDR2, and LATS1 to induce apoptosis [12, 13].

KIBRA was identified as a regulator of the Hippo pathway recently. In Drosophila, Kibra functions together with Mer (homolog merlin in mammals) and Ex in a protein complex and regulates the Hippo kinase cascade via direct binding to Hpo and Sav [14]. In addition to the regulation of MST1/2, KIBRA directly regulates LATS1/2 kinase activity by modulating the phosphorylation and stabilizes LATS2 by inhibiting its ubiquitination [15].

In this Review, we largely refer to Hippo signaling components using the mammalian nomenclature, and the *D*. *melanogaster* components are listed in Table 1.

**Table 1:** Post-translational modification controlling hippo pathway core components

Core component (*D. melanogaster* protein)	Enzyme	Regulatory sites	Function
MST1/2 (Hpo)	PI3K/Akt1	T120-p, T387-p	Prevention of caspase-mediated cleavage of MST1
	Autophosphorylation	T183-p^a^	MST activation
	JNK1	S82-p	Enhancement of MST1-mediated pro-apoptotic signaling
	mTOR	T120-p	Loss of MST1 function
	c-Abl	Y433-p	Inhibition of degradation
	TAO3^b^		MST activation
	PP2A	T183-dep^a^	
	CHIP	Ubiquitination	Protein degradation
SAV1 (Sav)	MST2	T26-p, S27-p, S36-p, S269-p	Activation
	SIK2 and SIK3	S413-p^c^	Antagonizing Hpo pathway activity
LATS1/2(Wts)	MST1/2	s909-p, T1079-p^d^	Enzymatic activity
	NUAK1	s464-p	Protein stability
	KIBRA	T1079-p^d^	Kinase activity
	Itch	Ubiquitination	Protein degradation
	CRL4^DCAF1^	Ubiquitination	Protein degradation
LATS1	WWP1	Ubiquitination	Protein degradation
	Nedd4	Ubiquitination	Protein degradation
LATS2	CHK1/2	S408-p	Cell cycle regulation, apoptosis
	SIAH1/2	Ubiquitination	Protein degradation
	Aurora A	S83-p, S380-p	Intracellular localization
MOB1A, MOB1B (Mats)	MTS1/2	T12-p, T35-p, T74-p	Molecular association
	praja2	Ubiquitination	Protein degradation
YAP (Yki)	LATS1/2	S61-p, S109-p, S164-p	The cytoplasmic retention
		S127-p	The binding of 14-3-3
		S397-p^e^	Facilitates the binding of CK1δ/ε, leading to further serine phosphorylations
	CK1ε/δ	S400-p, S403-p^f^	Generates a “phosphodegron” together with S397
	c-Abl	Y407-p^g^	Promotes p73-dependent pro-apoptotic transcription
	Src/YES1	Y407-p	Induces assembly of the β-catenin–YAP complex
	CDK1	T119-p, S289-p, S367-p	Cell cycle regulation
	NDR1/2	S127-p	The cytoplasmic retention
	JNK	T119-p, S138-p, T154-p, S317-p, T362-p	Inducing YAP to play dual role in different cell contexts
	SCF^β-TRCP^	Ubiquitination	Protein degradation
	Fbxw7	Ubiquitination	Protein degradation
	Set7	K494-me	Cytoplasmic retention
	CBP/p300	K494-ac; K497-ac	Altered nuclear activity
	PML	K97-sumo, K280-sumo^h^	Stabilizes YAP1 by sumoylation and enhances p73-dependent transcription
TAZ	LATS1/2	S66-p, S117-p	Cytoplasmic retention
		S89-p	The binding of 14-3-3
		S311-p	Facilitates the binding of CK1δ/ε, leading to further serine phosphorylations
	GSK3-beta	S58-p, S62-p	Degradation
	NEK1/CK1ε/δ	S314-p	Degradation/β-TrCP recruitment
	c-Abl	Y321-p	Altered nuclear activity
	PP1	S89-dep,S311-dep	Promotes TAZ nuclear translocation, and stabilizes TAZ by disrupting the binding to the SCF E3 ubiquitin ligase

**Fig. 2:**
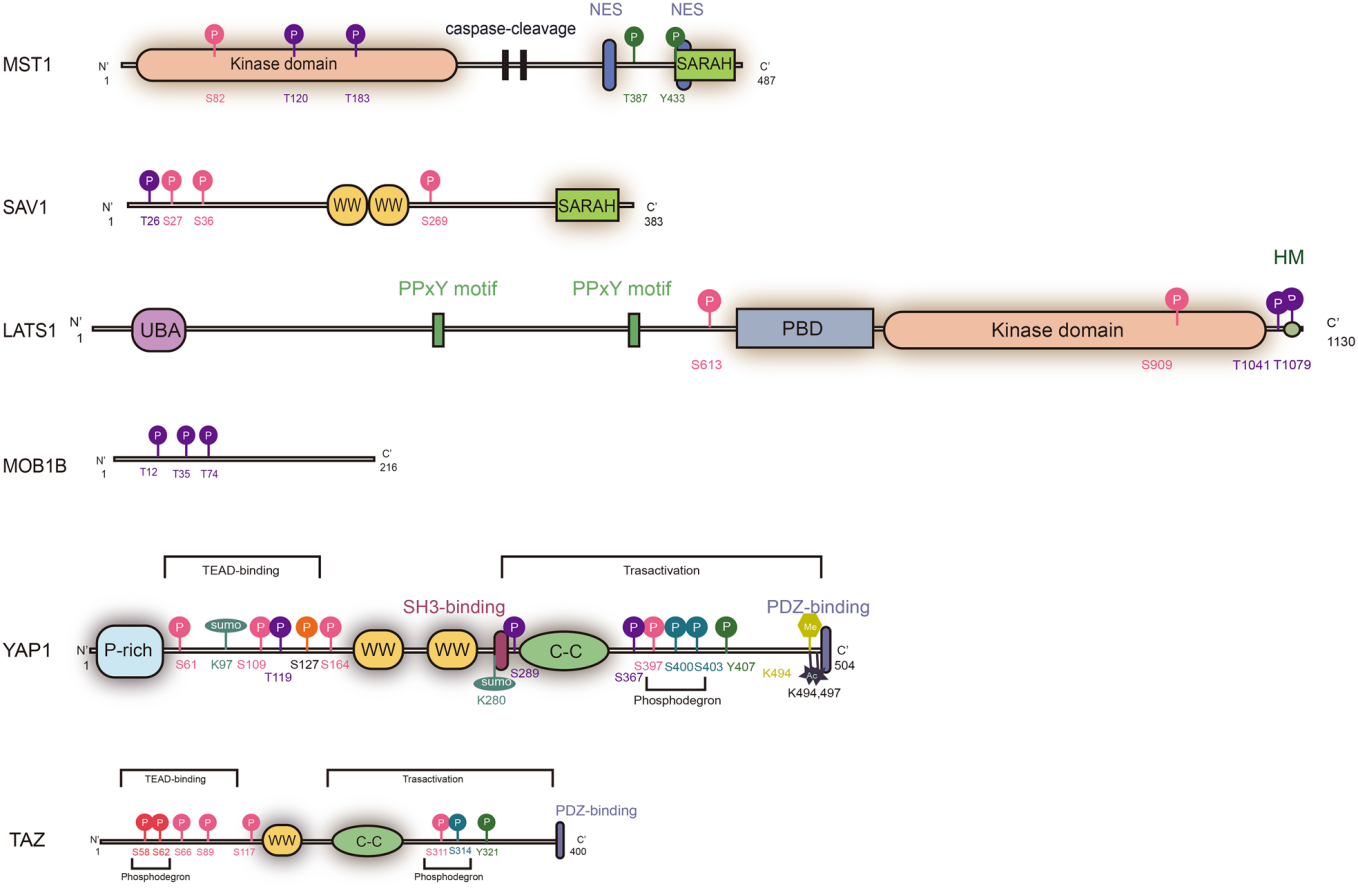
Schematic domain structure and PTMs sites of Hippo pathway core components. *SARAH* Sav/Rassf/Hpo domain; *NES* nuclear- exporting sequence; *WW* WW domain; *UBA* Ubiquitin-associated domain; *PBD* protein binding domain; *HM* hydrophobic motif; *P-rich* proline-rich region; *C–C* coiled-coil domain; *PDZ-binding* PDZ-binding motif. PTMs residue site details are listed in Table 1

The Hippo pathway acts primarily by inhibiting the nuclear functions of YAP and TAZ. Main PTMs of core components are described in Fig. 2; Table 1.)

*p* phosphorylation; *dep* dephosphorylation; *me* methylation; *ac* acetylation; *sumo* sumoylation

^a^ T180-p in MST2

^b^ Tao1 phosphorylates T195-p of Hpo

^c^ Phosphorylation of Sav at Ser 413 in Drosophila

^d^ Thr1079 in Lats1 and Thr1041 in Lats2

^e^ S381 of YAP1 iso2 corresponds to S397 of YAP1

^f^ S384, S387 of YAP1 iso2 corresponds to S400, S403 of YAP1

^g^ Y357 of YAP1 iso3 corresponds to S407 of YAP1

^h^ K242 of YAP1 iso3 corresponds to K280 of YAP1

### Post-translational modification of Hippo pathway

The Hippo pathway is considered to be in the active state when the MST and LATS kinases are active [16]. The MST1/2, aided by their regulatory protein SAV1, phosphorylates and activates the LATS kinases and MOB1A/B. The activated LATS1/2 in turn phosphorylates their downstream targets YAP and TAZ, leading to these two transcriptional co-activators in their nuclear export, cytoplasmic retention and/or proteasomal degradation. TEADs, without YAP and TAZ interaction, form complexes with the transcription cofactor vestigial-like protein 4 (VGL4), repressing target gene expression involved in cell proliferation. Thus, when the Hippo pathway is activated, YAP/TAZ activity is inhibited, and YAP/TAZ-driven gene expression is suppressed. Inversely, YAP and TAZ accumulate in the nucleus when the activity of upstream of Hippo pathway is inhibited, forming complexes with TEADs or other transcription factors. In this regard, the Hippo pathway acts primarily by inhibiting the nuclear functions of YAP and TAZ. Main PTMs of core components are described in Fig. 2; Table 1.

#### Phosphorylation

The fate of YAP/TAZ mainly decided by the phosphorylation status, determining their protein stability, activity and subcellular location.

LATS1/2 phosphorylate YAP at five serine residues (S61, S109, S127, S164 and S397) and TAZ has four of these sites (S66, S89, S117 and S311) [17]. Among these residues, S127 (S89 in TAZ) and S397 (S311 in TAZ) are key phosphorylation sites in suppressing YAP/TAZ oncogenic activity. The former is responsible for the spatial regulation (nucleo-cytoplasmic shuttling), while the latter is linked to the temporal regulation (degradation). Phosphorylation of S397 in YAP (S311 in TAZ) by LATS1/2 has been linked to regulation of protein stability, as it primes for the additional phosphorylation in YAP on S400 and possibly on S403 (S314 in TAZ) by CK1ε/δ, which generates a “phosphodegron motif” located in the C-terminal region of YAP/TAZ with pS397/pS311. This motif targets YAP/TAZ for polyubiquitination by recognizing and recruiting β-TrCP, a key adaptor of SCF E3 ubiquitin ligase, in this area and consequently facilitates proteasomal degradation. Being independent of LATS phosphorylation, GSK3-β phosphorylates of TAZ at S62, S58 in response to PI3K inhibition, finally forming N-terminal phosphodegron recognized by β-TrCP [18].

Phosphorylation of LATS also affect YAP/TAZ’s subcellular localization. Phosphorylation on S127 in YAP (S89 in TAZ) results in a binding site for 14-3-3 proteins, which would then keep YAP/TAZ in the cytoplasm. However, the sequestration of YAP/TAZ in the cytoplasm is not only achieved by S127 phosphorylation. Some observations indicate that there may be additional PTMs retaining YAP in the cytoplasm. Nuclear Dbf2-related kinases (NDR1/2) were found to phosphorylate S127 of YAP leading to the cytoplasmic retention and contribute to apoptosis and cell cycle regulation [19]. PP1A coupled with ASPP2 antagonizes the function of LATS to regulate the reversible phosphorylation of YAP/TAZ, increasing YAP/TAZ-dependent gene expression [20].

Apart from the classic LATS phosphorylation, YAP/TAZ are also targets of other kinases. JUN N-terminal kinases (JNK1 and JNK2) are identified as robust YAP kinases, with five novel phosphorylation sites on YAP identified: T119, S138, T154, S317 and T362 [21]. Upon UV irradiation, JNKs is stimulated to phosphorylate YAP. YAP to play a dual role in different cell contexts: YAP plays a protective role in keratinocytes but promotes UV-induced cell death in BWT skin squamous cell carcinomas, determining by the isoforms of p63 or p73 it binds. However, the same regulatory site, YAP pY357, plays two opposite roles based on the nature of the tyrosine kinase, either c-Abl or YES1. Cross-talking with Wnt/β-catenin pathway, YES1-mediated YAP modification is oncogenic and induces assembly of the β-catenin–YAP complex coactivating TBX5 target genes [22]. C-Abl, in the contrary, antagonizes the YAP oncogenic function, depresses YAP–TEAD-induced transcription and increases the binding affinity of YAP1 and p73, activating proapoptotic targets [23]. The nucleus dephosphatase PTPN14 could dephosphorylate YAP Y357 phosphorylation to induce cell proliferation [24]. In line, HIPK2 kinase regulates YAP abundance independent of its ability to regulate SCF^β-TrCP^ and in parallel to LATS1 and LATS2 to enhance YAP’s activity [25]. In addition, in mammalian cells, HIPK2 possibly influences YAP activity by promoting YAP abundance, whereas in *D. melanogaster* tissues, Hipk affects Yki localization.

Phosphorylation status of other core components are under regulation of kinases and phosphatases as well. In response to apoptotic or stress stimuli, MST1/2 is autophosphorylated at multiple sites in the activation loop, which results in the activation of MST1/2. Among these autophosphorylation sites, phosphorylation at T183 and T180 is essential for MST1 and MST2 activation, respectively [26]. These sites can also be trans-phosphorylated by the Ste20 family kinase TAO kinase-3 (TAO3), resulting in MST activation [27]. Besides autophosphorylation, MST1/2 are also regulated by trans-phosphorylation by protein kinases such as Akt, mTOR, JNK1, c-Abl. Akt binds to the C-terminus of MST1 and phosphorylates MST1 at the T120 and T387 residues. Phosphorylation of MST1 by Akt prevents caspase-mediated cleavage of MST1 and hence prevents activation of MST1 [28]. The mTOR signaling pathway also regulates MST1 phosphorylation at T120 and that this phosphorylation results in loss of MST1 function in prostate cancer cells [29]. Phosphorylation of MST1 on S82 by JNK1 enhances MST1-mediated pro-apoptotic signaling [30]. C-Abl inhibits the degradation of MST1 through CHIP and, secondly, triggers association between MST1 and FOXO3, thereby activating the MST1-FOXO signaling [31].

#### Ubiquitination

Besides the classical SCF^β-TrCP^ induced ubiquitination mentioned above, F-Box and WD repeat domain containing 7 (Fbxw7) was found to regulate YAP protein abundance by targeting YAP for ubiquitination and proteasomal degradation in hepatocyte carcinoma [32].

Notably, ubiquitination is more ubiquitous among the kinases of YAP/TAZ. Convincing evidence shows that the protein levels of LATS1/2 kinases are controlled by Itch E3 ubiquitin ligase-mediated degradation [33, 34]. Recent work shows that E3 ligase CRL4^DCAF1^-mediated inhibition of LATS1/2 in the nucleus is associated with Merlin/NF2 loss-driven tumorigenesis. Upon translocation into the nucleus, this form of Merlin binds to DCAF1 and suppresses CRL4^DCAF1^ activity, different from Merlin activity on core Hippo kinase cascade in the cytoplasm [35]. In addition, LATS1 and LATS2 are found to own their unique E3 ligases in different studies. Neuronally expressed developmentally downregulated 4 (Nedd4) and WWP1 E3 ligases inhibit the activity of LATS1 by targeting it for ubiquitin-mediated degradation, which turn out to promote tumorigenicity in different types of tumors, while SIAH1 and SIAH2 E3 ligases regulate LATS2’s stability in response to hypoxia [36, 37]. The co-factor of LATS1/2, MOB1, goes through proteolysis by E3 ligase praja2, which attenuates Hippo signaling and supports glioblastoma [38].

MST1 is also reported to be recognized by E3 ligase C terminus of Hsc70-interacting protein (CHIP). Inhibition of c-Abl promotes the degradation of MST1 through CHIP-mediated ubiquitination, and thereby attenuates cell death [39].

In contrast with E3 ligase, Deubiquitinases (DUBs) can remove monoubiquitin or polyubiquitin chain from the substrate, rescuing proteins from degradation fate or changing proteins’ activity state. CYLD is found to negatively regulate Hippo signaling by limiting Hpo phosphorylation at T195 in Drosophila, increasing Yki’s activity [40]. However, it remains to be determined whether the deubiquitinase activity of dCYLD is essential for limiting Hpo phosphorylation.

#### Sumoylation

Eleonora Lapi and colleagues find that PML and YAP physically interact through their PVPVY and WW domains, respectively, causing PML-mediated sumoylation and stabilization of YAP in a pro-apoptotic autoregulatory feedback loop between PML, YAP, and p73 [41].

#### Acetylation

Specifically, the YAP acetylation cycle is a balance between acetylation and deacetylation and is subjected to regulation by the transcriptional coactivator activity of human YAP. Nishita and colleagues provide important evidence that suggested YAP acetylation occurs on specific and highly conserved C-terminal lysine residues and is mediated by the nuclear acetyltransferases CREB binding protein (CBP) and p300 [42]. Conversely, the nuclear deacetylase NAD-dependent protein deacetylase sirtuin-1 (SIRT1) is responsible for YAP deacetylation. Intriguingly, this kind of YAP acetylation is induced specifically by S(N)2 alkylating agents and not by other DNA-damaging stimuli [43].

#### Protein methylation

Methylation of non-histone proteins is emerging as a regulatory mechanism to control protein function. Monomethylation of lysine 494 of Yap via Set7 is critical for cytoplasmic retention [44]. Thus this methylation-dependent checkpoint in the Hippo pathway may play a inhibitory role in YAP activation.

### PTMs of Hippo pathway in human cancers

The Hippo pathway regulated and is regulated by several cellular properties that are linked to tumorigenesis and progression. However, Hippo pathway genes are infrequently germline or somatically mutated in human cancers whether in targeted or whole genome sequencing research; this is especially true of the core Hippo pathway genes, example for neurofibromin 2 (NF2; also known as Merlin) in neurofibromatosis and malignant mesothelioma, and LATS2 in malignant mesothelioma. At the same time, the deregulation of the Hippo pathway has been reported at a high frequency in a broad range of different human carcinomas, including breast, lung, liver, colorectal, ovarian and prostate cancers, and it often correlates with poor patient prognosis. In light of these findings, we can imagine the probable pivotal role of PTMs of Hippo pathway in human cancers.

#### Breast cancer

Elevated TAZ expression is observed in more than 20 % of breast cancers, especially invasive ductal carcinomas. And overexpression of TAZ in “normal” mammary cell line MCF10A cells promotes cell proliferation, EMT, and oncogenesis. Mutation of the N-terminal phosphodegron created by GSK3-β will increases TAZ activity in inducing EMT in MCF10A cells [18].

Itch and WWP1 have been found negatively regulate LATS1 stability in breast cancer cells [33, 36]. Meanwhile, Ring-finger E3 ligase SIAH1, activated by HIF-1, is required for the hypoxia-induced ubiquitination and proteasome-dependent degradation of LATS2 and trigger nuclear localization of TAZ, leading to the breast cancer stem cells (BCSCs) maintenance. Similarly, SIAH2 promotes tumour growth through downregulation of LATS2 in breast cancer cells [37] and SIAH2 knockdown xenograft tumor growth is much suppressed.

#### Hepatocellular carcinoma (HCC)

Fbxw7 was found to regulate YAP protein abundance in HCC [32]. Fbxw7 expression was impaired in HCC tissues and loss of Fbxw7 expression was correlated with poor clinicopathological features.

The expression of SIRT1 is significantly upregulated in the HCC samples, and SIRT1 deacetylates YAP2 protein in HCC cells, which increases the YAP2/TEAD4 association, leading to YAP2/TEAD4 transcriptional activation and upregulated cell growth in HCC cells [43].

#### Nervous system tumor

In mesothelioma cells with NF2-mutant and derepression of CRL4^DCAF1^, cell proliferation, colony formation and tumor growth in xenograft mice will all be enhanced by inactivating LATS1/2 and, hence, activating YAP in the nucleus [35].

#### Hematological cancers

YAP was markedly downregulated in hematological malignancies, including lymphomas, leukemia and multiple myeloma [45]. In normal haematological cells, YAP1 forms a complex with the tumour p73 to support the transcription of proapoptotic genes, such as BAX and PIG3 expression [46]. In multiple myeloma cells, low expression of YAP prevents apoptosis in the presence of DNA damage in these haematological malignancies. Re-expression of YAP in multiple myeloma cells with YAP deletion or knockdown of MST1 in multiple myeloma cells with wild-type YAP, promoted apoptosis and growth arrest [45].

### Promising target and therapeutic implication

The PTM deregulation of Hippo pathway signaling in cancer triggers us to target these PTM elements in this pathway as an anticancer therapeutic strategy. Riskily and challengingly, identification of druggable components of these regulatory network will present opportunities for targeting therapy. For therapeutic strategies to be successful, the cancer must be functionally reliant on the pathway component that is being targeted.

#### Kinases and phosphatases

Targeting the kinase cascade of Hippo signaling could be by increasing the activities of MST1/2 and/or LATS1/2 kinases functioning upstream of YAP/TAZ. Instability of tumor suppressor kinases and depression of their expression in Hippo pathway promote downstream transcriptional co-activator YAP/TAZ to carry out their oncogenic roles in various tumors. The integrin-linked kinase (ILK) plays a role in suppressing the Hippo pathway by phospho-dependent inactivation of MYPT1 phosphatase leading to inactivation of Merlin/MST/LATS key components of the Hippo pathway [47]. Using ILK inhibitor QLT0267 in breast, prostate and colon tumour cells results in the activation of the Hippo pathway components MST1 and LATS1 with concomitant inactivation of YAP/TAZ and TEAD-mediated transcription. HIPKs and SIKs represent possible drug targets to blunt YAP/TAZ activity, as both kinases promote YAP activity in human cells [25, 48]. The G-protein coupled receptor (GPCR) has been reported recently link to Hippo-YAP/TAZ pathway [49]. Since many currently used therapeutic compounds target GPCR signaling directly or indirectly GPCR-Hippo signaling represents an attractive druggable target YAP/TAZ [50].

#### Ubiquitin–Proteasome system

To date, E3 ligase inhibitory compounds have been designed and discovered to have tumor suppression function, which will be listed as follows (Table 2).

**Table 2:** Compounds targeting Hippo pathway PTMs regulators

Target	Compounds	Function
ILK	QLT0267	Activation of the Hippo pathway components MST1 and LATS1 with concomitant inactivation of YAP/TAZ and TEAD-mediated transcription
Itch	Clomipramine	A clinically useful antidepressant drug. The effects in cancer cells treated with clomipramine included reduction of cell growth, and synergism with gemcitabine or mitomycin in killing cancer cells through autophagy blockade
Nedd4	Heclin	Bicyclic peptides, kills HEK293 cells growing in culture
SIAH1/2	BI-107F7	Peptide-mimetics, efficient covalent inhibitors of Siah and antagonize Siah-dependent regulation of Erk and Hif signaling in cell
	BI-107F9	

### -Itch

Clomipramine, a tricyclic antidepressant, has been identified as Itch inhibitor via a high throughput screen for small molecular weight inhibitors of the HECT E3 ligase [51]. Clomipramine can induce reduction of cancer cell growth, and synergism with gemcitabine or mitomycin in killing human breast, prostate and bladder cancer cells through autophagy blockade. However, Itch is known to target multiple proteins for ubiquitination, which enhances the complexity of predicting the consequence of Itch inhibition in vivo.

### -Nedd4

Amounting evidence has revealed that NEDD4 is frequently overexpressed in a range of human cancers which suggests that NEDD4 is a legitimate target for designing new drugs to treat human malignancies. Recently, A few bicyclic peptides isolated by phage display were demonstrated to have the capacity in targeting the E2 binding sites on the HECT domains of Smurf2, Nedd4, Mule/Huwe1, and WWP1, and act as specific inhibitors of these enzymes in vitro [52]. One of these peptides, Heclin, kills HEK293 cells growing in culture, consistent with an essential role for HECT ligase activity in mammalian cells.

### -Siah1/2

Proof-of-concept for small molecule inhibition of Siah has been obtained with a short protein fragment that binds competitively with high affinity to the substrate binding site of Siah proteins, and resulted in reduced growth of breast cancers and reduced frequency of metastases in melanoma. A rational structure-based design strategy was successful for the identification of novel Siah inhibitors BI-107F7 and BI-107F9, in which peptide binding drives specific covalent bond formation with the target [53]. X-ray crystallography, mass spectrometry and functional data demonstrate that these peptide-mimetics are efficient covalent inhibitors of Siah and antagonize Siah-dependent regulation of Erk and Hif signaling in cells.

## Conclusion and future prospects

Increasing evidence has attracted significant attention to the role of Hippo signaling in the developmental control as well as tumor initiation, invasion and drug resistance. In this review we summarized and discussed the importance of posttranslational modification such as phosphorylation, ubiquitylation, acetylation and protein methylation in orchestrating upstream signaling transduction, YAP/TAZ nuclear locational and transcriptional activation. Disrupted posttranslational modifications have been tightly connected to the aberrant development and different types of tumor formation. Emerging development of novel chemical inhibitors of various signaling molecules in the Hippo-pathway has shed light on next era strategy of anti-cancer therapeutics. In-depth exploration of the impact of posttranslational modifications in regulating Hippo signaling pathway opens a new avenue to advance the molecular mechanism of tumor initiation, invasion and occurrence, which complements well with the present techniques for diagnosis and therapeutics.

While previous efforts sketched a reasonable framework of posttranslational modification network in the regulation of Hippo signaling pathway, future work from the following aspects is necessary to establish a posttranslational modification regulatory circuitry in an entire shape. (1) The recent proteomic studies suggested possible crosstalk among various types of posttranslational modifications happen on the same protein that determines its functional. Thus the development of more sophisticated measurement of protein modifications and thorough dissection of integrated posttranslational modifications on the same signaling molecule such as YAP/TAZ will enhance our mechanistic understanding. (2) Despite the discovery of the role for a series of modification enzymes such as ubiquitin protein ligase, acetyltransferase, protein methyltransferase and protein kinase, studies for the counteracting enzymes in balancing the posttranslational modifications such as deubiquitinase, deacetyltransferase, demethyltransferase as well as phosphatase need to be carried out. (3) Bioinformatics in data mining of TCGA analysis with mutations on the protein modifiers or critical site on the substrates that facilitates the protein modification will lead to the foundation of the personalized strategy to combat cancer. (4) Development of specific antibodies that recognize specific modification sites could result in new biomarkers of early diagnosis. (5) Development of inhibitors that prevent the abnormal posttranslational modifications could be utilized for chemosensitization or precision therapeutics.

## Abbreviations

BCSCs: breast cancer stem cells
CBP: CREB binding protein
CHIP: C terminus of Hsc70-interacting protein
DUBs: deubiquitinases
Fbxw7: F-box and WD repeat domain containing 7
GPCR: G-protein coupled receptor
HCC: hepatocellular carcinoma
ILK: integrin-linked kinase
JNK: JUN N- terminal kinase
KIBRA: protein kidney and brain expressed protein
LATS: large tumor suppressor
MOB1A: MOB kinase activator 1A
MST: mammalian Ste20-like kinases
NDR: nuclear Dbf2-related kinases
Nedd4: neuronally expressed developmentally downregulated 4
NF2: neurofibromin 2
PTMs: posttranslational modifications
RASSFs: RAS association domain- containing family proteins
RUNX1: RUNT-related transcription factor 1
SAV1: salvador homolog 1
SIRT1: NAD-dependent protein deacetylase sirtuin-1
TAO3: TAO kinase-3
TBX5: T-box transcription factor 5
TEAD: transcriptional enhancer factor TEFs
VGL4: vestigial-like protein 4
YAP: yes-associated protein
